# Wakefulness

**Published:** 1876-09

**Authors:** 


*TWENTY-THIRD YEAR OF*

### HALL'S JOURNAL OF HEALTH.

*E. H. Gibbs, M.D., Editor.*

VOL. XXIII.] SEPTEMBER, 1876. [NO. 9.

### *WAKEFULNESS.*

This is a prevalent complaint, and, when persistent, requires careful attention and judicious treatment. For, although not immediately dangerous, its continuance during any considerable time, is certain to result disastrously to both mind and body. It is frequently followed by inflammation of the brain, or paralysis, and it almost always precedes insanity. Yet, by all except the sufferer, it is usually regarded as one of the minor ills of life, and we listen with impatience to the story of the weary vigils that make life a burden.

It would be difficult to define the causes of wakefulness. They are as numerous and as varied as the emotions that sway the human heart. Whatever disturbs the mental or physical equilibrium, induces an unnatural condition of the nervous system. Wakefulness frequently arises from causes affecting the mind, while stupor—a still more alarming disease—may be induced by the same causes, or may proceed from bodily injury, such as concussion of the brain, or loss of blood. A rule with few exceptions is: that death from depletion, as from loss of blood, or from copious purging, or in cholera, is preceded by stupor and coma. While death resulting from depriving the system of what is necessary to sustain life—food and water—is preceded by delirium. But, as before stated, there are exceptions to this rule, and two persons may suffer from diseases, to all appearance exactly the opposite, but induced by the same causes. Why this is so, is one of those mysteries that we are constantly encountering, but shall probably never be able to solve. Fortunately, its solution is not so important now as it was a few years ago, when physicians made indiscriminate use of the lancet in all such cases. While cases of sleeplessness that are induced by care and anxiety are always the most serious, and demand our greatest solicitude, their number is, fortunately, few in comparison with those that are induced by causes that might be easily removed. These are irregular hours, imprudence in eating and drinking, and in the use of tobacco, and want of attention to the condition of the bowels. It would be useless to offer any advice to that class who suffer from causes, perhaps, beyond their own control. Many of these, however, would be benefited, some probably cured, by careful attention to their bodily health. What we mean by "regular hours," is not that a person should go to bed at sunset, and get up at sunrise; that would be simply "routine," an impossibility for men engaged in the active affairs of life.

We believe that every person should have at least eight hours rest and sleep out of every twenty-four hours, to be taken at any time that it may be required, and that may be most convenient.

The ability to sleep depends very much on the will and determination of the person. We are acquainted with a gentleman who has entire faith in the power of the will over sleep. He said that for years he was troubled with wakefulness; not from care or worry, but on account of the habit he had fallen into after retiring to bed, of planning his work for the next day. He tried various remedies without avail, until his health became greatly impaired. There seemed but one thing left that he could try with any hope of success, and that was the power of his will to keep his mind quiet. To use his own language--"whenever his thoughts began to come," he said, "go away, I can't attend to you," and placing himself in a comfortable position, he would close his eyes, and refuse to allow his thoughts to take any definite shape. Since adopting this plan and persevering in it, he has had no trouble to sleep whenever he desires.

By imprudence in eating and drinking, we do not mean, altogether, imprudence in eating and drinking too much. There are extremes in all things. But where one person has been injured by an excess of wholesome food, a hundred have been destroyed for the want of it. Half the sickness, and more than half of the unhappiness in the world, is caused, indirectly, perhaps, by insufficient and unwholesome food.

A medical friend of ours remarked that the kindliest sympathies of our nature were certain to be found in the hearts of men possessing generous stomachs, or, as he expressed it—"ample pantry room."

Such men seldom suffer for want of sleep; and we never saw a fat lunatic. We have been consulted by many sufferers from wakefulness, whose troubles we have traced to insufficient nourishment, and in no instance has the trouble continued after the fault was corrected. The pernicious effects of tobacco on the nervous system are too well understood to require further notice in this place. The use of strong green tea, particularly in the evening, frequently prevents sleep. Whenever it is found that it induces wakefulness, its use should be given up. Coffee is not so apt to excite the nerves as green tea ; still should it do so, its use should be suspended.

There is hardly a disease where constipation is not present, and usually as the most prominent element for evil. In the disease that we are considering, it is often the chief cause of the trouble. The present effects of constipation are restlessness and discomfort, and its ultimate effects are destructive of health and life. It is a disease that indirectly destroys more lives than any other. It strikes in every direction by poisoning the blood on which the integrity of the system depends, and by which every part is nourished and sustained. Whatever the disease, the first inquiry of the educated physician is in relation to the condition of the bowels ; and his first care is to secure their regular action ; and this must be preliminary to all other treatment. Many cases of wakefulness are caused by constipation alone, and there is no further trouble after daily action of the bowels is established. Purgatives should never be given for this purpose. They only afford temporary relief, and result in permanent injury. Injections are almost equally objectionable. Fortunately, we need never be compelled to resort to either. The "Suppository"—we prefer, "Nelaton's,"—has never failed in our practice to accomplish all that we could desire, and its effects are permanently beneficial.

